# DOK1 facilitates the advancement of ccRCC

**DOI:** 10.7150/jca.104375

**Published:** 2024-10-14

**Authors:** Wei Xie, Yuanfeng Zhang, Bian Shu, Zhechuan Zhang, Ronggui Zhang

**Affiliations:** 1 The Affiliated Chongqing General Hospital of Chongqing University, School of Medicine, Chongqing University, Chongqing, China, zip code: 401147.; 2 Department of Urology, The Second Affiliated Hospital of Chongqing Medical University, Chongqing, China, zip code: 400010.; 3 Department of Hepatobiliary Surgery, The Second Affiliated Hospital of Chongqing Medical University, Chongqing, China, zip code: 400010.

**Keywords:** DOK1, PI3K-AKT, ccRCC, epithelial-mesenchymal transition, KIRC

## Abstract

**Background:** Renal cell carcinoma (RCC) is one of the most common human cancers. Clear cell renal cell carcinoma (ccRCC) is a major subtype of RCC. However, the molecular mechanisms underlying ccRCC oncogenesis require further investigation. Docking protein 1 (DOK1) is a putative tumor suppressor gene; however, its role in ccRCC remains unclear.

**Methods**: Bioinformatic analysis was used to illustrate the poor prognosis associated with DOK1 expression and its role in tumor development in ccRCC in patients. qPCR (quantitative polymerase chain reaction) and western blotting assays were used to validate DOK1 expression in ccRCC cells. *In vitro* experiments were performed to further elucidate the biological role of DOK1 in ccRCC.

**Results:** DOK1 was overexpressed in ccRCC tissues and cells at both mRNA and protein levels. High DOK1 expression closely correlated with poor survival in patients with ccRCC. DOK1 expression significantly accelerated ccRCC proliferation, migration, invasion, and epithelial-mesenchymal transition (EMT). Through PI3K (phosphatidylin-ositol-3-kinase)/AKT (protein kinase B)/GSK3β (glycogen synthase kinase 3 beta) signaling, DOK1 may control the progression of ccRCC.

**Conclusion:** DOK1 has the potential to serve as a valuable biomarker and target for treatment in ccRCC through its regulation of PI3K/AKT/GSK3β signaling to promote ccRCC progression.

## 1. Introduction

Renal cell carcinoma (RCC) is a commonly occurring and fatal type of cancer 1. Approximately 80% of kidney cancers are clear cell renal cell carcinomas (the main subtype of RCC; ccRCC) 2. Distinct vitreous cytoplasm has been identified as the main histological feature of ccRCC 3. In patients with RCC, surgery can lead to improved progression-free survival; however, 30% patients develop tumor cell metastasis after surgery 4. Advancements in targeted therapies, including mTOR inhibitors and immune checkpoints, have revolutionized the treatment for RCC 5. Resistance often arises owing to the diverse and continuously changing characteristics of the tumor microenvironment (TME) 6. Drug tolerance and metastasis are the two major adverse effects associated with ccRCC patient outcomes 7. It is crucial to gain deeper insight into the fundamental processes of ccRCC to discover diagnostic and prognostic markers and identify novel targets for treatment.

Different types of human tumors frequently exhibit rearranged locus of *Docking protein 1* on chromosome 2p13.1 8. Additionally, the biological function of DOK1 has been studied mainly in the hematopoietic system, and multiple studies have indicated that DOK1 is positively correlated with the oncogenesis of several hematopoietic malignancies, including chronic myelogenous leukemia (CML) 8, chronic lymphocytic leukemia (CLL) 9, histiocytic sarcoma 10, and Burkitt's lymphoma 11. However, the biological functions of DOK1 in ccRCC have been poorly studied. Therefore, multilevel analyses, including bioinformatics and *in vitro* experiments, were performed to examine the biological role of DOK1 in ccRCC. Based on this, DOK1 may serve as a potential therapeutic target for ccRCC in the future.

DOK1 overexpression in ccRCC indicated poor survival. Furthermore, DOK1 silencing reduced ccRCC proliferation and metastasis. Notably, the changes triggered upon DOK1 knockdown were primarily mediated by PI3K/AKT signaling. We have demonstrated the tumor promoting role of DOK1 in ccRCC.

## 2. Materials and Methods

### 2.1 Bioinformatic analysis

Clinical information of patient samples and RNA-seq data were obtained from The Cancer Genome Atlas (TCGA), which consisted of 72 paracancerous tissues and 537 kidney renal clear cell carcinoma (KIRC) tissues. The GSE535757 dataset was downloaded to supplement the KIRC data from TCGA. Furthermore, survival, univariate, and multivariate Cox analyses were performed based on the downloaded RNA-seq data and clinical data from TCGA database. R software was used to analyze differentially expressed genes (DEGs) and for gene ontology (GO) (Figure S1A and B) and KEGG analyses 12,13. Additionally, protein levels of DOK1 in KIRC and paracancerous tissues were investigated using the Human Protein Atlas (HPA) online database.

### 2.2 Cell culture

We cultured five ccRCC cell lines, 769-P, OSRC2, 786-O, A498, CAKi-1, and one healthy cell line, HK2, in an incubator using RPMI-1640 (Gibco) containing 10% fetal bovine serum at 37 °C with 5% CO2.

The OSRC2 and 786-O cell lines were procured from Pricell. The cell lines were authenticated using GeneMapper IDX software and compared with the ATCC, DSMZ, JCRB, and Cellosaurus databases. In addition, the two cell lines were tested for the presence of *Mycoplasma*. Details of cell lines are provided in the Supplementary Information.

### 2.3 *In vitro* short interfering RNA (siRNA) transfection

Cells were seeded in 6-well plates and cultured until the cell density reached approximately 70%. Cells were then transfected with 100 pmol siRNA (Tsingke), 125 µL Opti-MEM (Gibco), and 4 µL Lipo-8000 (Beyotime) per well. Six hours later, fresh medium was added. Reverse-transcription quantitative PCR (qRT-PCR, 48 hours later) and western blotting (96 hours later) were performed to validate the efficiency of DOK1 knockdown. The RNAi sequences used were as follows: #1 (sense (S), CCCUGAACCUGGUACUGCA; antisense (A), UGCAGUACCAGGUUCAGGG), #2 (S, CCAUCUAUGAUGAACCUGA; A, UCAGGUUCAUCAUAGAUGG), #3 (S, GAGGGAGUACAACGGAAGA; A, UCUUCCGUUGUACUCCCUC), and NC (S, UUCUCCGAACGUGUCACGU; A, ACGUGACACGUUCGGAGAA).

### 2.4 Cell proliferation

After transfection, ccRCC cells were seeded in 96-well plates and cultured for 24-72 hours to examine proliferation. Twenty-four hours after culture multiple sets of (0,1,2,3) cells were treated with CCK-8 (APExBIO, USA) for 2 hours at 37 °C Thermo. Absorbance was measured at 450 nm using a LUX Multimode Microplate Reader (Marchine). EdU proliferation assay was performed to detect cell proliferation. Initially, cells were seeded into 24-well plates and cultured at 37 °C with 5% CO2. The Cell-Light EdU Apollo *In Vitro* Kit (RIBOBIO) was used according to the manufacturer's instructions. An inverted Nikon fluorescence microscope was used to capture the images.

### 2.5 Scratch and transwell assays

Changes in the migration of ccRCC cells were examined using a wound-healing assay. Next, ccRCC cells were cultured in 6-well plates until they attained approximately 90% confluence. Then, the monolayer was scratched using a 200 μL pipette tip, and the medium was changed to RPMI-1640. Cell scratch images were obtained at 0 and 24 h, using an inverted microscope.

Metastasis of ccRCC cells was analyzed using a Transwell assay. For migration assays, 500 µL complete medium was added to each well of 24-well plates, and the transwell chambers (each chamber with 200 μL medium containing 4 × 10^3^ ccRCC cells) which were then placed in 24-well plates. After 12 hours of culture at 37 °C, cotton buds were used to wipe off the remaining cells. Images were acquired using an inverted microscope at room temperature after the chambers were immersed in 4% multiformaldehyde for 20 min and stained using 0.5% crystal violet for 30 min. For the invasion assay, Matrigel was placed in Transwell chambers, and the same procedure was performed as that used in the migration assay.

### 2.6 qRT-PCR

Using the SteadyPure Quick RNA Extraction Kit (Agbio), t-RNA (Total RNA) was extracted from ccRCC cells using the SteadyPure Quick RNA Extraction Kit (Agbio). We synthesized cDNA using the PrimeScript® RT reagent and amplified with the CFX Connect system with the SYBR GreenTM Premix Ex Taq II (Takara) according to the manufacturer's instructions. β-actin was used as a control. The primers used were as follows: β-actin, forward (F), 5' ACA ACT TTG GTA TCG TGG AAG G-3', reverse (R), 5'-GCC ATC ACG CCA CAG TTT C-3'; DOK1, F, 5'-CAA TTC TGG GTA ACG GTG CAG-3', R, 5'-CCA CCC TCA GCA CGT AGG A-3'.

### 2.7 Western blotting

Approximately 20-40 μg total protein lysates obtained from ccRCC cells were separated via SDS-PAGE using the 10% PAGE Gel Fast Preparation Kit (Epizyme, China), and electro-transferred onto PVDF membranes. Membranes were blocked with the Protein-Free Rapid Blocking Buffer (Epizyme) prior to overnight incubation with primary antibodies, and then incubated with the secondary antibody for 60 min. Antibodies were procured as follows: Anti-β-Actin (Cat#113225, Servicebio), Anti-DOK1 (Cat#17822-1-AP, Proteintech), Anti-E-cadherin (Cat#20874-1-AP, Proteintech), Anti-Vimentin (Cat#60330-1-Ig, Proteintech), Anti-Snail (Cat#13099-1-AP, Proteintech), Anti-Slug (Cat#9585T, CST), Anti-N-cadherin (Cat#22018-1-AP, Proteintech), Anti-AKT1/2/3 (Cat#T55561S, Abmart), Anti-PI3K (Cat#4257T, CST), Anti-GSK-3β (Cat#ab32391, Abcam), Anti-pAKT1/2/3 (Cat#ab192623, Abcam), Anti-pPI3K (Cat#GTX132597, Gene Tex), Anti-pGSK-3β (Cat#310010, ZENBIO). Proteins were identified using enhanced chemiluminescence (ECL) and the CLINX ChemiScope S6 (Shanghai) (SS1701, Zhongguan).

### 2.8 Statistical analysis

Experiments were repeated three times, and the GraphPad Prism 8 software was used to plot the results and analyze the differences between two groups (Student's *t*-test). Statistical significance was set at p values < 0.05.

## 3. Results

### 3.1 DOK1 is overexpressed in ccRCC

**Figure 1:**
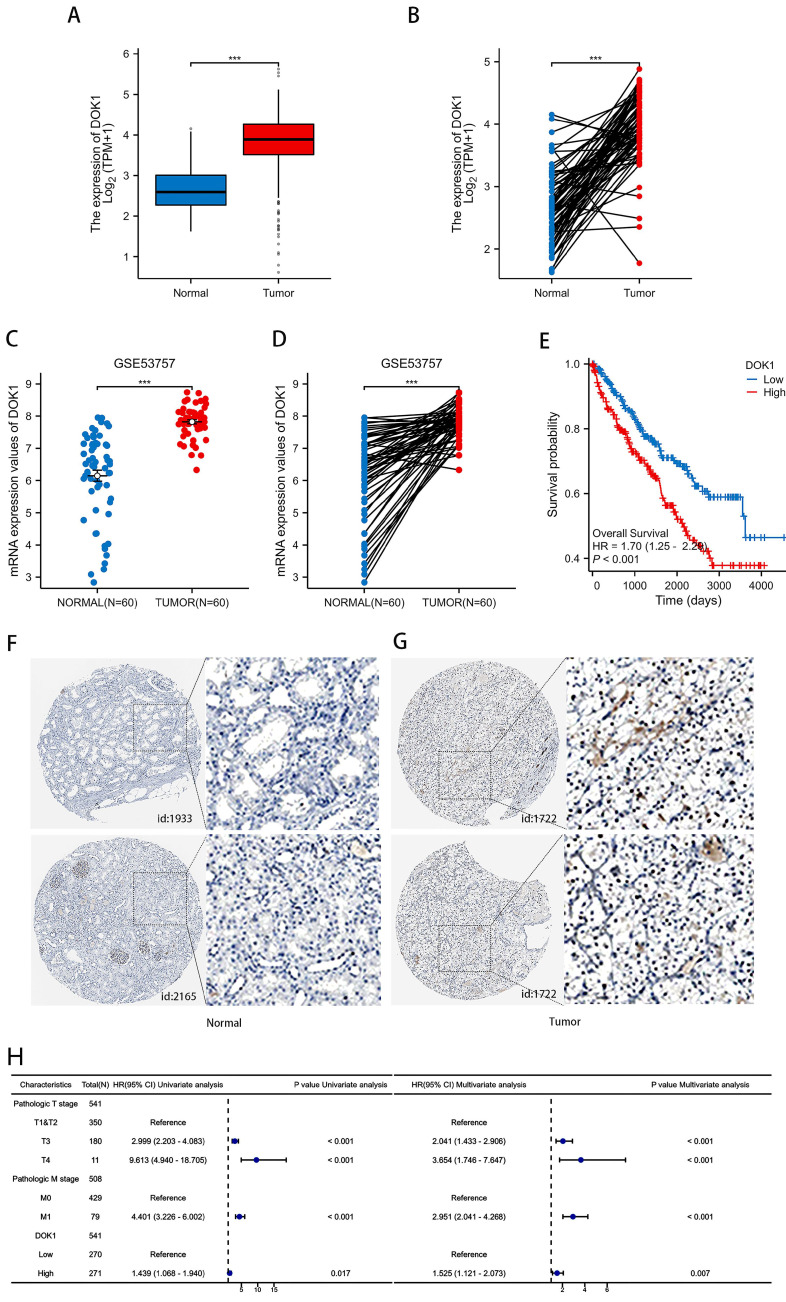
** DOK1 overexpression predicts poor ccRCC prognosis.** (A and B) DOK1 expression detected using the TCGA database. (C and D) DOK1 expression in cancerous and paracancerous tissues using unpaired (C) and paired tests (D) found using the GSE53757 dataset. (E) Upregulated DOK1 predicts poor survival as shown by results from the TCGA database. (F and G) Visualization of DOK1 using IHC in ccRCC (G) and healthy tissues (F), the results for kidney tissues obtained using the HPA online database. (H) Univariate and multivariate Cox analyses for DOK1 in ccRCC. (*p < 0.05, **p < 0.01, ***p < 0.001).

Changes in DOK1 expression in ccRCC tissues were first examined to investigate its biological role in progression of the disease. As presented in Figure 1A and 1B, DOK1 was strongly upregulated in ccRCC tissues compared to healthy tissues in the KIRC cohort, both, in unpaired and paired comparisons. Furthermore, patients with ccRCC with high DOK1 expression revealed positive correlation with poor overall survival (Figure 1E). Univariate and multivariate Cox analyses showed that DOK1 may be an independent risk factor for ccRCC. We further analyzed the GSE53757 dataset (Figure 1C and D). Consistent with this the HPA database revealed that patients with ccRCC showed strong DOK1 expression (Figure 1F). These results confirmed that upregulation of DOK1 expression might be involved in ccRCC oncogenesis and indicate poor overall survival.

### 3.2 DOK1 silencing inhibits ccRCC proliferation and metastasis

**Figure 2:**
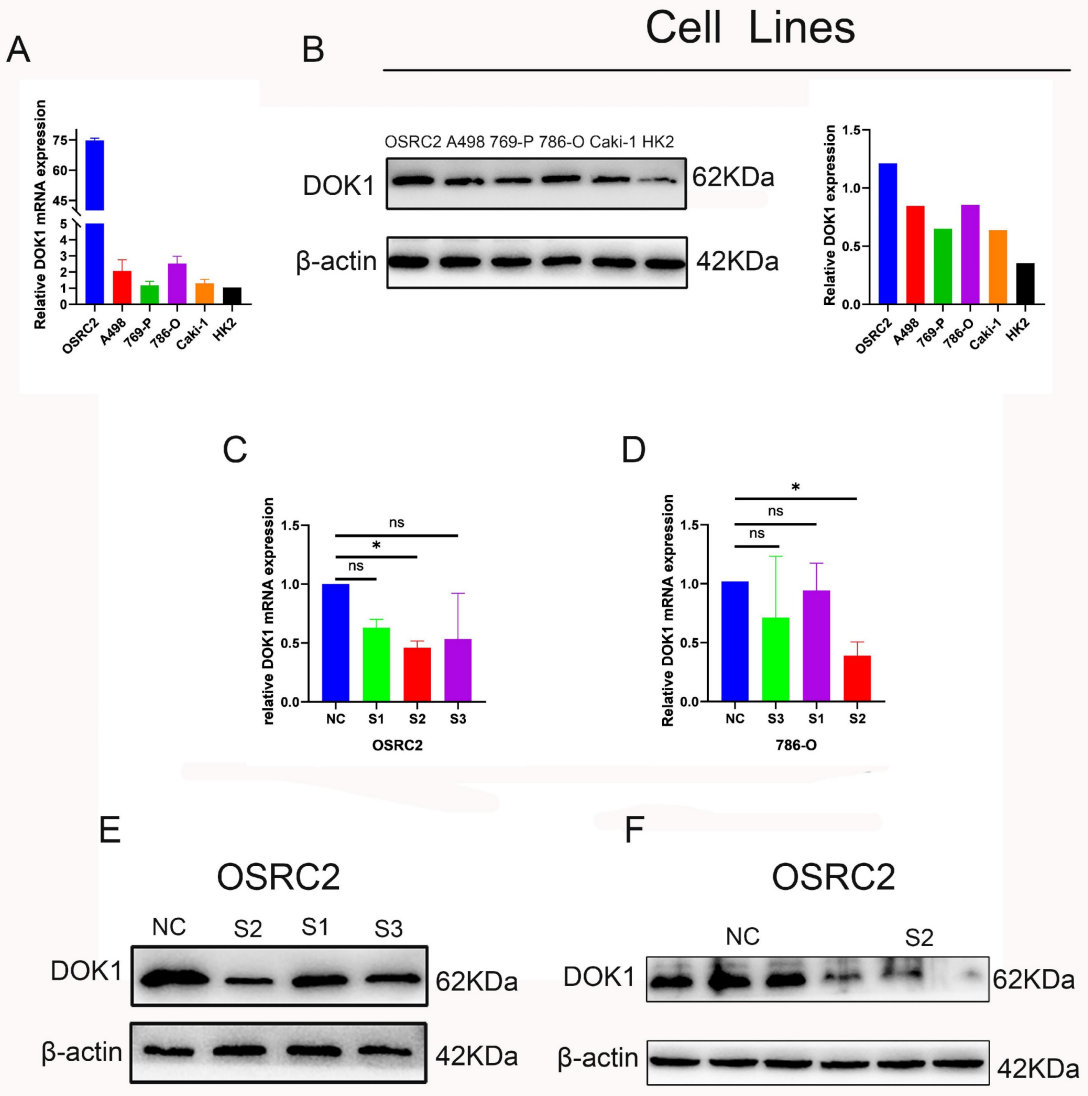
** Determination of DOK1 expression in ccRCC cell lines and DOK1 knockdown efficiency.** (A and B) qRT-PCR (A) and western blotting (B) were used to determine mRNA and protein levels of DOK1 in ccRCC cell lines. (C and D) qRT-PCR results showing knockdown efficiency of DOK1 in OSRC2 (C) and 786-O (D) cells. (E and F) western blotting showing siRNA II knockdown efficiency in OSRC2. (*p < 0.05, **p < 0.01, ***p < 0.001).

**Figure 3:**
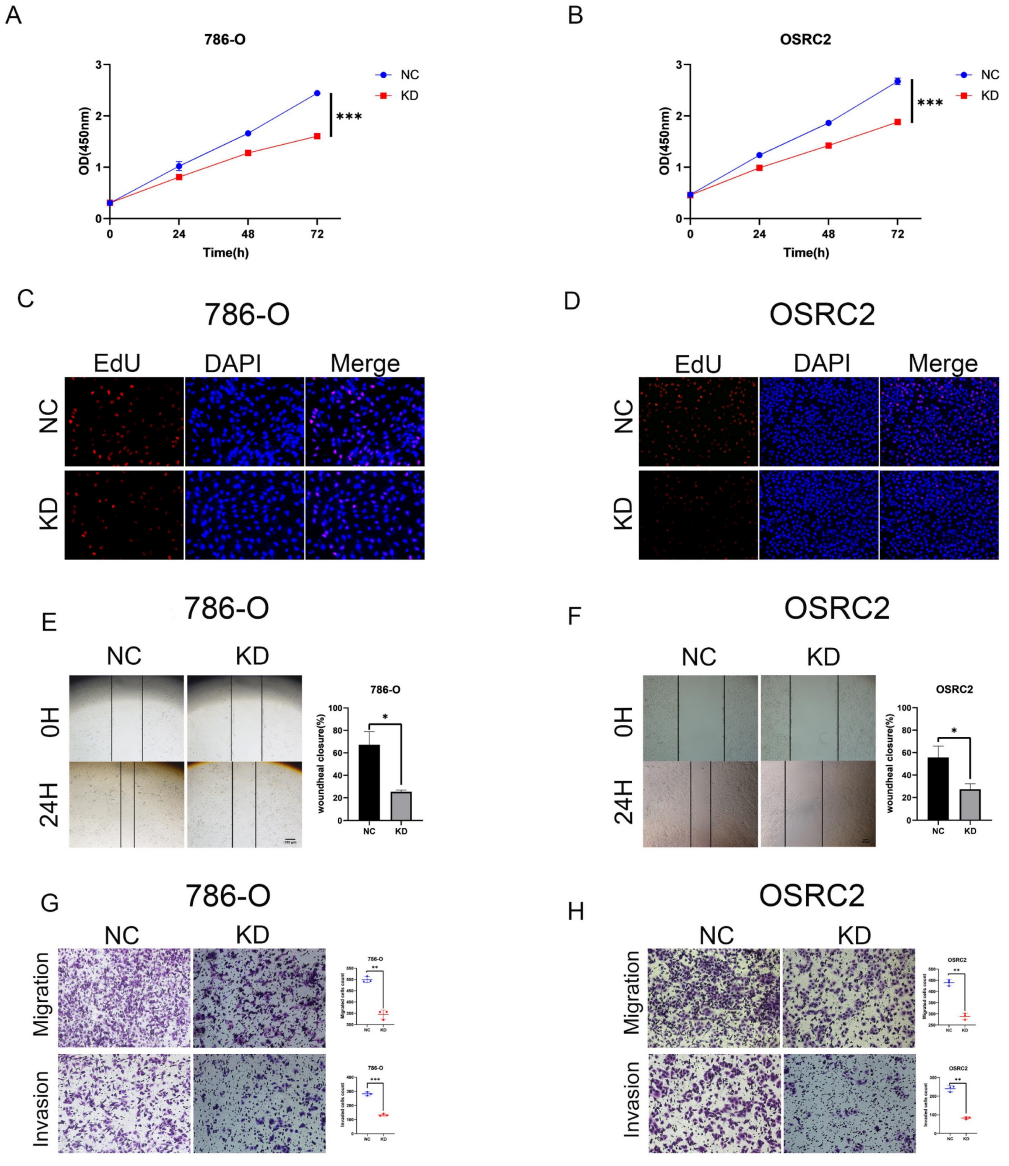
** DOK1 accelerates ccRCC cell proliferation and metastasis.** (A and B) CCK-8 assays were used to determine 786-O (A) and OSRC2 (B) cells proliferation. (C and D) EdU assays were used to further verify 786-O (C) and OSRC2 (D) cell proliferation (×200). (E and F) Wound healing assays were used to show 786-O (E) and OSRC2 (F) cell migration. (G and H) Transwell assays showing 786-O (G) and OSRC2 (H) cell migration and invasion (×100). Bar = 100 μm. (*p < 0.05, **p < 0.01, ***p < 0.001).

We performed several *in vitro* experiments to further investigate the role of DOK1 in ccRCC progression. Using RT-PCR and western blotting we verified results from five ccRCC cell lines (e.g., Caki-1, 786-O, 769-P, A498, and OSRC2) and one healthy kidney cell line (HK2) (Figure 2A and B). Results revealed that OSRC2 and 786-O cell lines showed high expression of DOK1 compared with that seen in the HK2 cell line. We designed three siRNAs to knockdown DOK1 in ccRCC cells, which were transiently transfected and validated these results using RT-PCR and western blotting (Figure 2C- F). Further, we found that DOK1 silencing inhibited ccRCC cell proliferation using CCK-8 (Figure 3A and B) and EdU proliferation assays (Fig. 3C and D). In addition, wound healing assays (Figure 3E and F) and transwell assays (Figure 3G and H) were performed using ccRCC cells to study the potential biological role of DOK1 in migration and invasion. These results showed that knockdown of DOK1 inhibited the migration and invasion of 786-O and OSRC2 cells, indicating that DOK1 silencing restrains proliferation and metastasis of ccRCC cells.

### 3.3 DOK1 knockdown inhibits EMT in ccRCC

**Figure 4:**
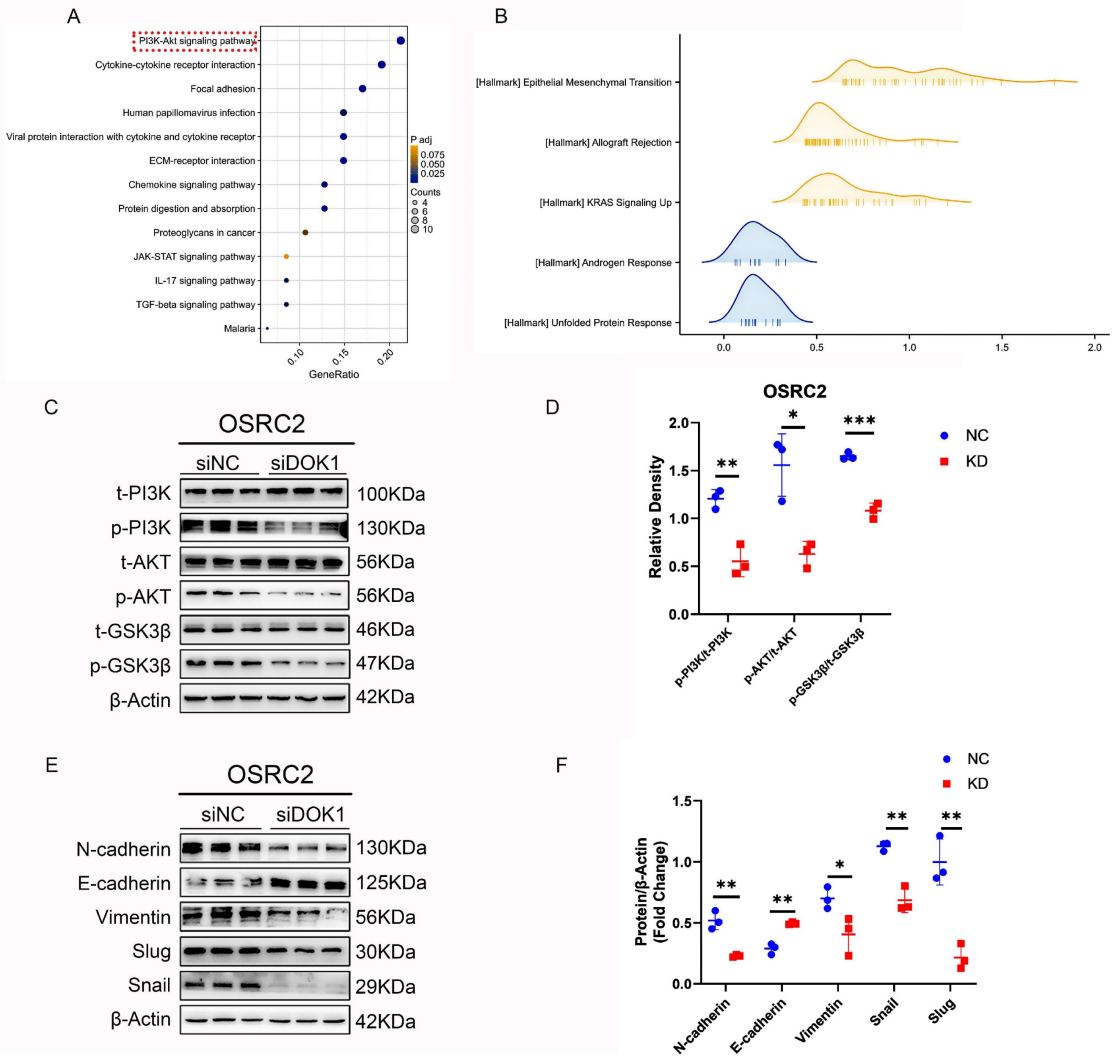
** DOK1 promotes EMT by regulating PI3K-AKT-GSK3β signaling in ccRCC.** (A and B) KEGG analysis (A) and hallmark of GSEA (B) of upregulated DEGs. (C and D) Western blotting results showing PI3K, p-PI3K, AKT, p-AKT, GSK3β, and p-GSK3β protein levels. (E and F) Western blotting results showing EMT markers. (*p < 0.05, **p < 0.01, ***p < 0.001).

To understand the oncogenic role of DOK1 in ccRCC, a differential expression gene analysis was performed using the TCGA database data. Additionally, we used GSEA to demonstrate the role of DOK1 in ccRCC, and results indicated that DOK1 was positively and closely correlated with EMT (Figure 4B). In addition, western blotting was performed to investigate whether EMT markers (e.g., N-cadherin, E-cadherin, Vimentin, Snail and Slug) were altered upon DOK1 knockdown *in vitro*. We concluded that DOK1 was involved in EMT in ccRCC cells (Figure 4E and F).

### 3.4 DOK1 might be involved in the PI3K/AKT/GSK-3β signaling pathway in ccRCC

Based on data obtained from TCGA, KEGG enrichment analysis (Figure 4A and Figure S1C) was performed to determine the signaling pathway via which DOK1 is involved in ccRCC. Based on these results, we hypothesized that DOK1 is involved in PI3K/AKT signaling (Fig. 4A).

Furthermore, western blot results confirmed that DOK1 silencing decreased PI3K and AKT phosphorylation (p-PI3K and p-AKT) without affecting their total levels (Figure 4C and D). These findings indicated that DOK1 promotes ccRCC progression by regulating PI3K/AKT signaling. In addition, GSK3-β total and phosphorylation (p-GSK-3β) levels have also been validated using western blotting assays as a well-known molecular mechanism downstream of the PI3K/AKT signaling pathway (Figure 4C and D). Notably, DOK1 silencing showed similar effects on GSK-3β with PI3K and AKT. We concluded that DOK1 may regulate the progression of ccRCC via the PI3K/AKT/GSK-3β signaling pathway.

## Discussion

DOK1 is considered to have antitumor roles in several cancers, such as chronic lymphocytic leukemia (CLL) 9, chronic myelogenous leukemia (CML) 8, histiocytic sarcoma 10, lung cancer 14, and epithelial ovarian cancer 15. Currently, little is known about ccRCC. In this study, we discovered the reverse function of DOK1 in CML. DOK1 upregulation is closely associated with poor survival in ccRCC, and DOK1 may be an independent risk factor for ccRCC. DOK1 levels were observed to be highly elevated in ccRCC cell lines. In addition, siRNA-mediated DOK1 silencing inhibited ccRCC cell proliferation. Moreover, ccRCC cell migration and invasion were restrained upon DOK1 knockdown. Bioinformatic assays indicated that the PI3K/AKT signaling pathway is a vital molecular mechanism via which the biological role of DOK1 is involved in the progression of clear cell renal cell carcinoma. Moreover, DOK1 silencing significantly decreased p-PI3K and p-AKT levels. GSK-3β is the downstream component of the PI3K/AKT signaling pathway. Thus, we then verified whether p-GSK-3β levels altered upon DOK1 knockdown. Western blotting results revealed that GSK-3β phosphorylation also reduced upon DOK1 silencing. These results indicate that DOK1 could have a significant impact on ccRCC through regulation of the PI3K/AKT/GSK-3β pathway.

**Figure 5:**
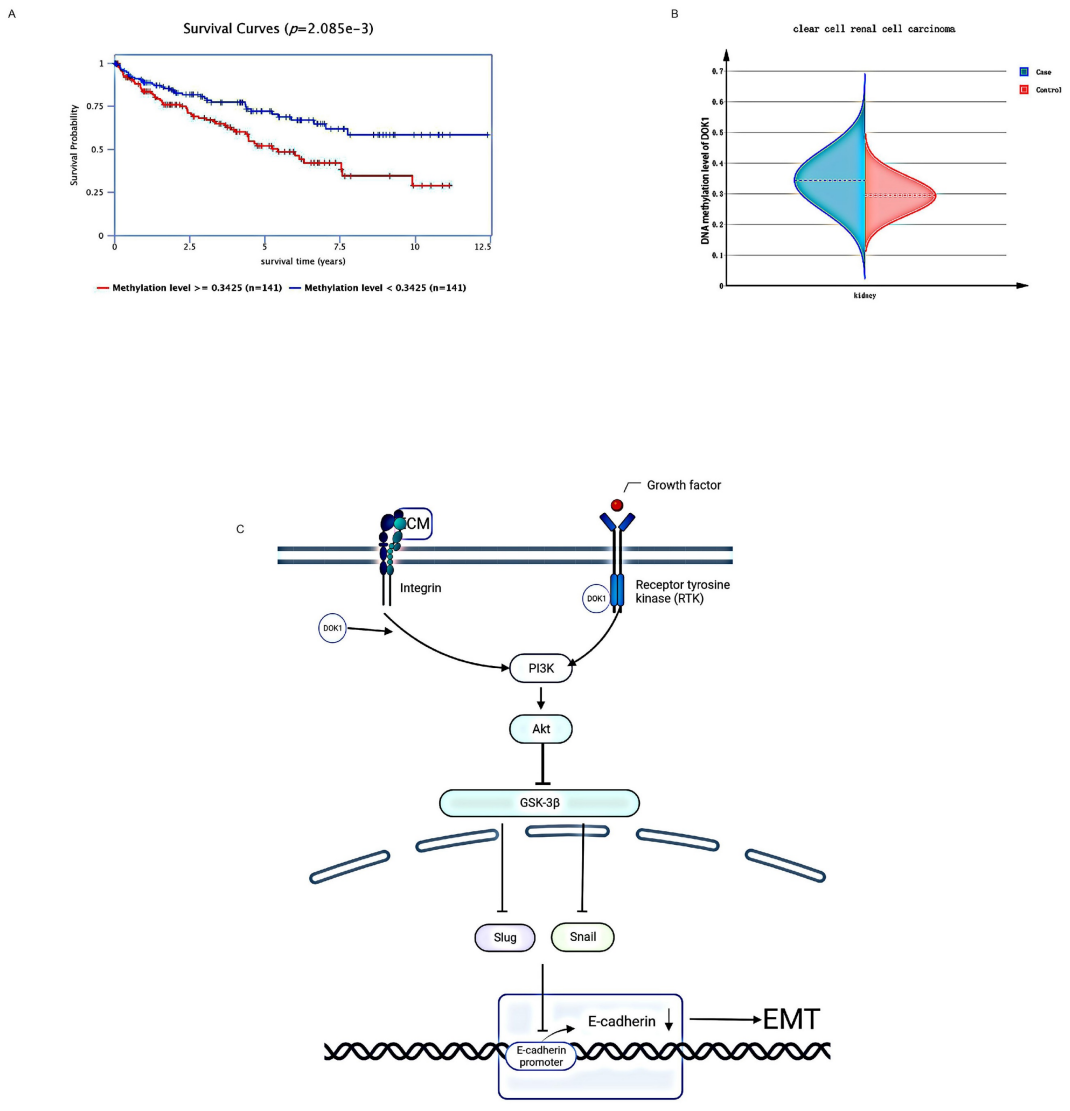
** DOK1 may target the PI3K/AKT/GSK3β pathway to regulate ccRCC EMT, and methylation of DOK1 revealed poor prognosis.** (A and B) DOK1 showed high methylation in ccRCC tissues. (C) DOK1 may be a promising candidate molecule regulating EMT in ccRCC via regulation of the PI3K/AKT/GSK3β signaling pathway.

In accordance with the GSEA results, we observed that DOK1 expression was positively correlated with EMT. We then performed western blotting to examine EMT-linked proteins. Results indicated that DOK1 knockdown significantly reduced Slug and Snail expression, however, increased E-cadherin expression. Multiple studies have reported that Slug and Snail play important roles as transcription factors in the EMT process 16-18. In addition, numerous studies have revealed that Snail and Slug play important roles in regulating EMT and metastasis in ccRCC 19-23. In recent studies, Snail has been confirmed as a key molecule in regulating p-GSK-3β levels 24-27. Moreover, the expression and stability of Slug can also be regulated by p-GSK-3β 28. In addition, GSK-3β is involved in the PI3K/AKT signaling pathway; thus, p-GSK-3β can be regulated via this signaling pathway. We concluded that DOK1 may regulate EMT in ccRCC by targeting the PI3K/AKT/GSK-3β signaling pathway (Figure 5C).

Studies have shown that frequent hypermethylation of DOK1 plays a significant biological role in the progression of malignant cells and in development of cancerous characteristics of various types of human tumors 29. Therefore, we speculated that DOK1 expressed in ccRCC may be methylated to promote ccRCC progression. The Epigenome-Wide Association Study (EWAS) Data Hub was used to determine DOK1 methylation in ccRCC. Compared to those seen in healthy tissues, DOK1 methylation levels in ccRCC tissues were significantly higher (Fig. 5B), and high methylation levels of DOK1 predicted a poor prognosis (Fig. 5A) in patients with ccRCC. Accordingly, we concluded that the oncogenic function of DOK1 in ccRCC may be mediated by its methylation.

We revealed the regulatory role of DOK1 in ccRCC progression; however, this study has some limitations. First, the detailed mechanism of DOK1-mediated tumorigenesis in ccRCC and other human tumors remains unclear. Second, the conclusions of this study are based on *in vitro* experimental results and have not been verified using *in vivo* experiments. Additionally, the role of DOK1 was predicted using online databases, indicating that more local clinical data are required to verify these findings.

## Conclusion

We confirmed that DOK1 expression closely correlates with poor prognosis in ccRCC. Our findings revealed that DOK1 knockdown inhibits ccRCC proliferation and metastasis by blocking PI3K/AKT/GSK-3β signaling. In conclusion, DOK1 might be a promising therapeutic target for ccRCC.

## Supplementary Material

Supplementary figure.
