# Supplementary material for: DOK1 facilitates the advancement of ccRCC
Source: J Cancer. 2024 Oct 14;15(19):6213–22. doi: 10.7150/jca.104375 (PMC11540502; doi:10.7150/jca.104375)
Supplement: Supplementary file 1 — Supplementary figure. [file jcav15p6213s1.pdf]

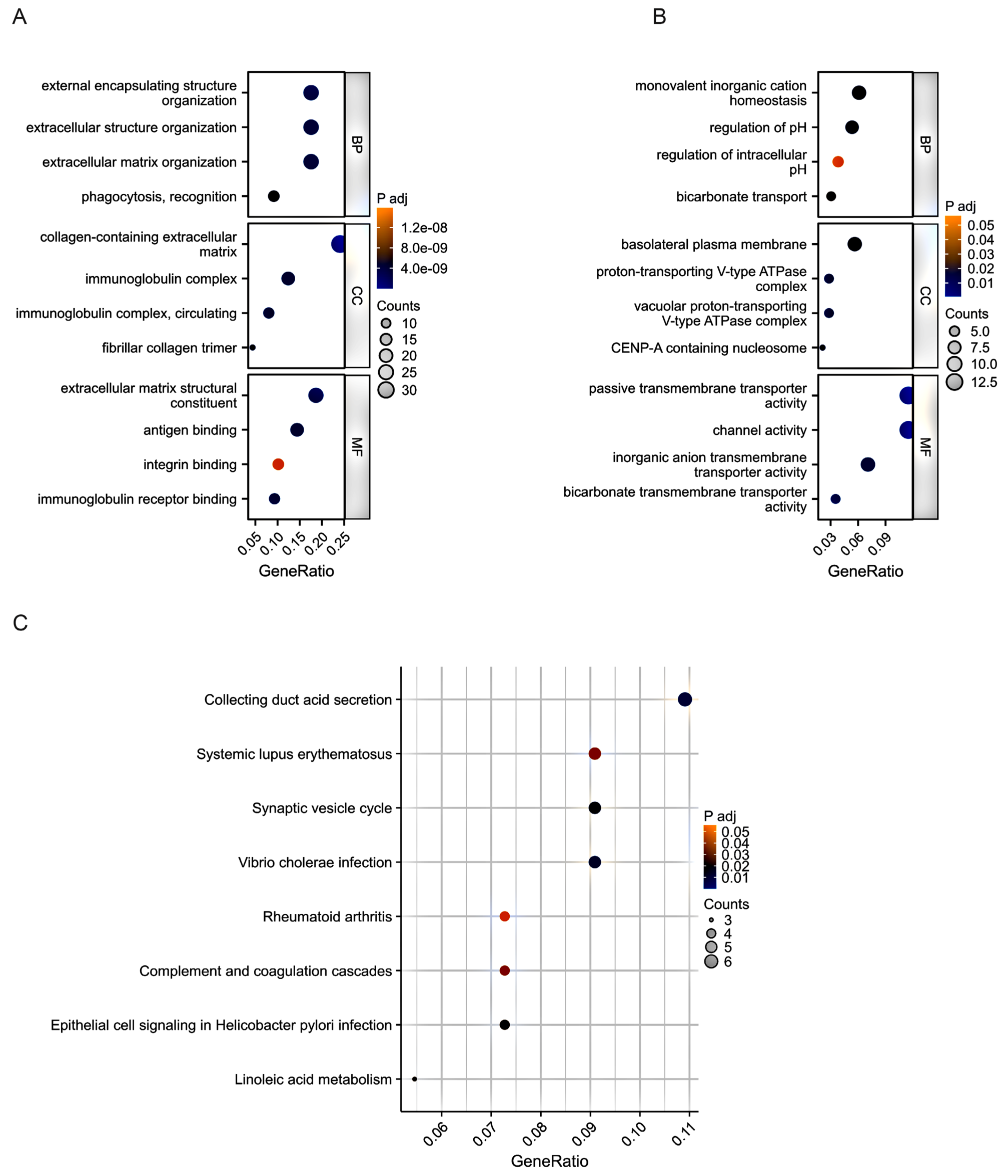

Figure S1. Enrichment analysis of the DEGs. (A and B) GO analysis of the DEGs. (C) KEGG analysis of the upregulated DEGs upon DOK1 knockdown.
